# Development of a values-based decision aid to determine discharge destination: Case reports of older stroke survivors and their families

**DOI:** 10.1097/MD.0000000000030934

**Published:** 2022-10-14

**Authors:** Yoriko Aoki, Kazuhiro Nakayama

**Affiliations:** a Department of Gerontological Nursing, Faculty of Medicine, University of Toyama, Toyama-shi, Toyama-ken, Japan; b Graduate School of Nursing Science, St. Luke’s International University, Chuo-ku, Tokyo, Japan.

**Keywords:** case report, decision aid, discharge destination, older stroke survivor

## Abstract

Older adults affected by stroke must face a difficult choice between receiving post-discharge care at home or in a facility. This study aimed to develop a decision aid (DA) to help older adults and their families choose the place of post-discharge care based on their values.

**Methods:**

Values and data for designing the DA were obtained through interviews with older stroke patients and their families, a questionnaire survey of various health professionals, and a review of patients’ medical records. Next, a prototypic DA was prepared and tested for comprehensibility and usability using the 12-item International Patient Decision Aid Standards instrument.

**Results:**

The DA was developed based on the following 6 values that were common among older stroke patients and their families: “activities of daily living,” “services and fees,” “emergencies,” “family support,” “environment,” and “home renovation.” The prototype met the criteria in the comprehensibility and usability tests.

**Conclusion:**

Older stroke patients can use the DA to think through the evidence-based information matching their own values to make a more satisfactory decision. The effectiveness of this DA should further be investigated in clinical settings.

## 1. Introduction

Cerebral stroke is the second leading cause of death worldwide.^[1]^ The morbidity and recurrence of stroke increase with age, and stroke is prone to aggravation. Therefore, older adults affected by stroke are faced with the difficult decision of whether to live at home as before or receive care elsewhere after discharge. Self-determination is not a written or codified right in Japan, and the majority of older patients make the decision with their families. Furthermore, as families are often responsible for taking care of the older members, the family’s say is prioritized in the decision-making process.^[2]^ In fact, it is not all rare that decisions about an older patient are made by other family members and medical and/or welfare professionals without the patient’s participation.

For this reason, studies on discharge support for older adults in Japan have mainly consisted of case studies and surveys from the perspective of families or professionals, such as identification screening items and developing screening tools for promoting early discharge,^[3]^ factors affecting the discharge destination,^[4,5]^ and nurses’ coordination abilities or decision-making support.^[6]^ There are almost no studies or assessments from the viewpoint of older adults. Decision-making support about discharge destination is one of the most difficult challenges in hospitals. Indeed, it has been reported that the greatest challenge is coordination to find a compromise between different intentions of older adults and their families and even professionals.^[7]^ However, an established method of decision-making support is not available for use in healthcare settings in Japan.

A very large number of randomized controlled trials have been included in reviews on discharge support for older adults in other countries.^[8–10]^ The benefits of discharge support evaluated in these studies were mainly related to costs, such as shortened hospital stays, lower readmission rates, decreased mortality, and increased satisfaction and quality of life of patients and caregivers. Pioneering studies on decision-making support have been conducted in the US, Canada, the UK, and Germany, and have reported that the involvement of the patient in decision-making^[11]^ and clarification of values are keys to better decision-making. Decision aids (DAs) are being developed actively as means of supporting decision-making and have been demonstrated to be effective for increasing knowledge, reducing conflicts and unclarity of values, and increasing participation in the decision-making process.^[12]^ van Weert et al^[13]^ showed that DAs are effective even in older adults for increasing knowledge, accurately perceiving risks, reducing conflicts in decision-making, and increasing patient participation. Evidence and the effectiveness of DAs for decision-making on where older adults should receive care have not yet been demonstrated because such decisions are particularly difficult to make due to the communication difficulties older adults face and the various social systems and people/places involved.^[14,15]^ To date, only Garvelink et al^[14]^ have developed a DA for selecting care at home or at a long-term care facility for frail older adults from the perspective of caregivers. However, participation by older adults in the development of this DA was difficult because they were frail or in the terminal stage.

In Japan, information brochures are commonly used for decision-making on discharge destinations, but they often contain more information than necessary and confuse many older adults and their families. This problem prompted us to develop a DA in which the information is arranged to help older adults and their families compare the pros and cons of each option and make choices matching their values. However, only a small number of DAs have been developed previously in Japan, and none of them have been developed based on assessments by professionals in various disciplines. In addition, frail subjects such as older adults have very rarely been involved in the development of DAs anywhere in the world. This study thus aimed to develop a DA to help older adults and their families to make decisions on the place for post-discharge care based on their values.

## 2. Methods

### 2.1. Study design and process of development

**Figure 1.**
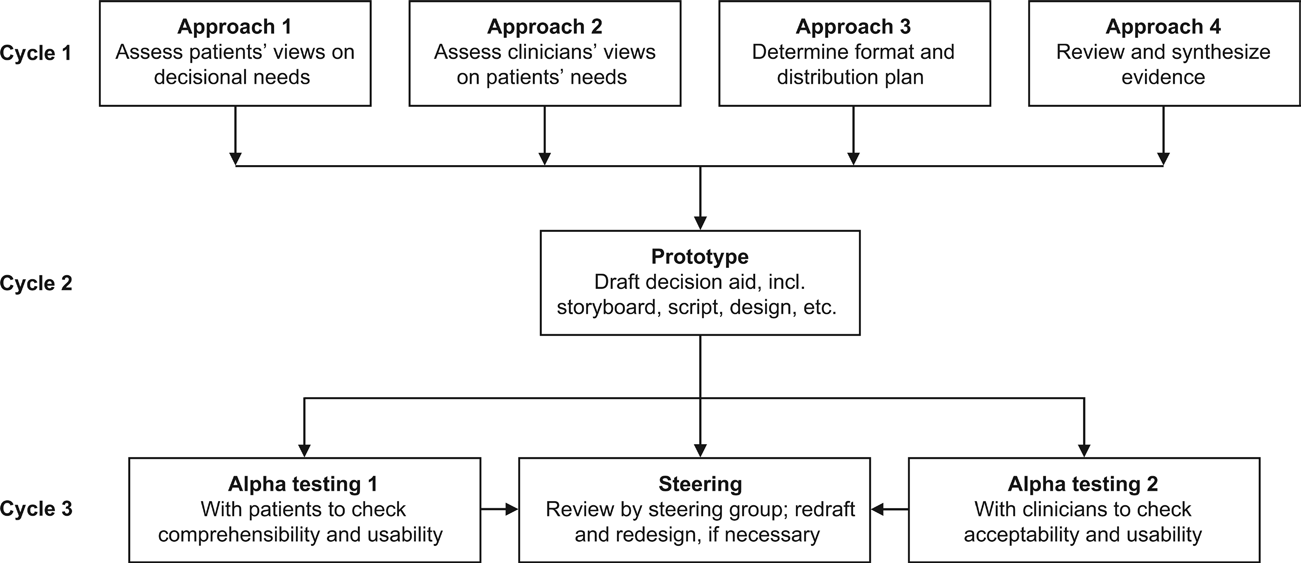
Model development process for decision aid.

This study consisted of a 3-cycle mixed quantitative/qualitative study involving older stroke patients, the ultimate users of the DA, families, and multidisciplinary clinicians (physicians, nurses, physical therapist [PT] occupational therapists [OT], and medical social worker [MSW]) to develop a decision-making guide in line with the values of older stroke patients and their families. Specifically, the steps consisted of Cycle 1: Selection of items, Cycle 2: The development prototype, and Cycle 3: Validity assessment. The process of development followed the structural process of Coulter et al,^[16]^ who performed a systemic review of articles on DA (Fig. 1), with references to the International Patient Decision Aid Standards instrument (IPDASi),^[17]^ Ottawa Personal Decision Guide,^[18]^ and DA for older adults^[14,19]^ as well as brochures issued by the Japanese local government units, such as cities, towns, and villages.

### 2.2. Cycle 1: Selection of items

#### 2.2..1. Approach 1. Assess patients’ views on decisional needs.

Convenience sampling was conducted on 10 older stroke patients aged ≥ 65 years and 10 key persons in their families. Those who could not clearly state their own intentions due to severe dementia or aphasia were excluded. We interviewed them about their values and needs associated with decision-making, and the data obtained from the interviews were analyzed.^[20]^

#### 2.2..2. Approach 2. Assess clinicians’ views on patients’ needs.

In total, 39 clinicians (7 physicians, 11 nurses, 8 PT, 4 OT, and 9 MSW) who were in charge of 10 older stroke patients who had been hospitalized for 1 week were surveyed. A questionnaire was developed to examine information items that patients and their families should know when deciding where to be discharged from the hospital, based on previous studies.^[3–5]^ We analyzed whether the information items were necessary or unnecessary by simple tabulation. Specifically, the survey inquired on items related to medical care (neurovascular disease): that is, disease, physical function (abilities and disabilities in activities of daily living [ADL]), cognitive decline, chronic medical care, medication management, family’s long-term care-giving capacities, and items related to public health and welfare: that is, service points, procedures of application for the physical disability certificate, need for and details of long-term care insurance (steps from application to certification, details and conditions for available services, fees, and list of providers) in a free response format. Respondents were further asked to select from one of the options as to who needed the above information for deciding discharge destination: patient only, family only, patient and the family, and not needed at all.

#### 2.2..3. Approach 3. Determine format and distribution.

Effective layout and format of the brochure were investigated by taking into account the advanced age of the older stroke patients and the characteristics of their common disorders and disabilities, such as motor disability, memory impairment, attention deficit, and aphasia.

#### 2.2..4. Approach 4. Review and synthesize evidence.

To incorporate the benefits and risks of each place for post-discharge care for older stroke patients in the DA, we referred to the 278-item Ottawa Hospital Research Institute A to Z Inventory^[21]^ to first research about existing and general DA. DAs with unclear details were ordered by contacting the authors directly via email, and a literature review was conducted using international databases, such as the Cochrane Library, Medline, CINAHL, and JAMAS, for Japanese articles only. The literature review was limited to academic papers, and a full-year search was conducted. The keywords used included “decision aids,” “location of care,” and “discharge planning,” and the age category was set to ≥ 65 years. In addition, “Stoke” was multiplied, and content related to mental illness and acute conditions was excluded.

### 2.3. Cycle 2: The development prototype

After accomplishing the Approach 1, 2, 3, and 4 stages, a prototype DA consisting of 12 A4-sized pages was prepared. Previous DA studies relied on the generic Ottawa Personal Decision Guide^[18]^ founded on the Ottawa Decision Support Framework (ODSF) as reference and also met the conditions of the IPDASi. DA derived from the ODSF can enhance knowledge and the accurate awareness of risk, promote decision-making matching personal values, and reduce the percentage of patients facing difficulty in making the decision; thus, using the ODSF is reported to be a valid approach for developing a DA.^[22]^ Furthermore, the Ottawa Personal Decision Guide incorporates data specialized for decision-making related to options, risks, and benefits, meeting the minimal quality standards of a DA.^[18]^ Therefore, we decided to apply the structure of introduction, facts and information, pros and cons of various options, expressing and weighing values, and checking preparedness for decision-making.

### 2.4. Cycle 3: Validity assessment

To meet the minimal scientific standards of a DA, 11 experts (5 researchers in decision-making and 6 graduate students who were nurses and public health nurses) were recruited to perform the comprehensibility test questionnaire to evaluate whether the DA met all the 12 minimal criteria of the IPDASi (Table 1), which entailed 6 items on qualifying criteria that determine that an intervention is a form of DA and 6 items on criteria to evaluate the risk of any detrimental biases, in the case that the qualifying criteria are not met. The qualifying criteria were evaluated binarily by a “yes” or “no.” The certifying criteria were evaluated on a scale from “not at all (1 point)” to “very much (4 points).” All qualifying criteria must be obligatorily met, but it is recommended that the certifying criteria be met as well.

**Table 1:** Comprehensibility test.

**Qualifying criteria**	1 DA describes health condition or problem for which index decision is required
	2 DA explicitly states the decision that needs to be considered (index decision)
	3 DA describes the options available for the index decision
	4 DA describes the positive features (benefits or advantages) of each option
	5 DA describes the negative features (harms, side effects, or disadvantages) of each option
	6 DA describes what it is like to experience the consequences of the options
**Certification criteria**	7 DA shows the negative and positive features of options in equal detail
	8 DA provides citations to the selected evidence
	9 DA provides a production or a publication date
	10 DA provides information about the update policy
	11 DA provides information about the levels of uncertainty associated with event or outcome probabilities
	12 DA provides information about the funding source used for development

IPDAS minimal qualifying and certification criteria for decision aids (Garvelink, 2016).

DA = decision aid.

Furthermore, a usability test (Table 2) was conducted with a questionnaire to evaluate the ease of using the DA on 20 subjects, including 1 older stroke patient aged ≥ 65 years, 1 family member, clinicians (1 physician, 1 nurse, 1 PT, 1 OT, and 1 MSW), 1 discharge support nurse, 1 older adult participant from the general population, and 11 experts in decision-making. The questionnaire included 7 items evaluated on a scale of “Not at all (0 points)” to “very much (3 points).” The questionnaire was created with reference to a previous study^[14]^ and the User Manual for Acceptability,^[23]^ and the respondents were encouraged to offer opinions for a draft DA in the free response section of the questionnaire.

**Table 2:** Usability test (N = 21).

**Designing**	1 Is the language in the decision aid understandable?
	2 Are you satisfied with the length of the decision aid (12 pages)?
	3 Is the presentation of the decision aid right for its target group and purpose (lay-out, size, font size, use of pictures)?
	4 Does the decision aid provide you with enough information?
	5 Is it clear how the decision aid should be used?
	6 Do you think the decision aid would be helpful for seniors and their caregivers who are in the process of decision-making about the location of care?
	7 Do you think the contents are biased toward “home,” “residence/facility (for those who are independent)” or “residence/facility (for those who need care)”?
**Acceptability**	8 What decisions need to be made and by when? (p.1–2)
	9 Gain the necessary knowledge (p.3)
	10 Learn about the “characteristics of each discharge destination” (p.4–6)
	11 Compare the advantages and disadvantages of each discharge location (p.7–8)
	12 How ready are you to decide? (p.9–10)

Reference: Garvelink (2016).

DA = decision aid.

### 2.5. Steering: Review by steering group; redraft and redesign, if necessary

The content was corrected and the layout was modified according to the results of the comprehensibility and usability tests to revise the draft DA. The layout was prepared by a graphic designer. Finally, the content of the completed DA was evaluated by older stroke patients, their families, and clinicians.

### 2.6. Ethics approval and consent to participate

Written informed consent to participate in the interview survey was obtained after explaining the nature of the study orally and in writing. Returned questionnaires were interpreted as consent for questionnaires distributed to clinicians and for evaluating content validity. This study was approved by the institutional review boards of St. Luke’s International University (16-A024), Toyama Prefectural Rehabilitation Hospital & Support Center for Children with Disabilities (No.31), and University of Toyama (28-63).

## 3. Results

### 3.1. Cycle 1: Selection of items

In cycle 1, the selection of items was conducted. First, as a result of the literature review, we found a total of 127 references, 72 in Japanese and 55 in English. There were 2 existing DAs on where to receive post-discharge care.^[14,24]^ However, they targeted end-of-life and frail older adults, and none addressed stroke patients. There were 2 Cochrane reviews on the location of post-discharge care for older stroke patients. Ward et al^[25]^ compared the benefits of home and hospital for the rehabilitation of older adults, and Boland et al^[26]^ compared home with other locations for the care of older adults, but the pros and cons of the location for post-discharge care of older adults were not mentioned clearly. That is, the 2 existing DAs did not provide evidence related to the place of convalescence, so rather than presenting the advantages and disadvantages, they had incorporated things the user should know and made references to preferences and values. Through this review, we found evidence that research on where older stroke patients decide to receive post-discharge care is not established, and we thus decided to review the medical records of 103 older stroke patients aged ≥ 65 years in the participating facilities to investigate the factors influencing discharge destinations. The results showed that the factors that influenced the discharge destination were “ADL,” “eating disorder,” and “family’s preference for discharge destination.”

Five values were identified: “Degree of independence in ADL,” “environment for convalescence,” “family relationship,” “disease management,” and “social resources and fees.” These were common values that affected the decision-making of older stroke patients and their families regarding the discharge destination (Table 3).

**Table 3:** Qualitative synthesis.

Category	Subcategory	
	Values of older adults	Values of the families
**Independence in activities of daily living**	Physical recovery (4)	Physical recovery (10)
	Residual disability (5)	Transfer/mobility (4)
	Ability to open envelopes for oral drugs (1)	Meals (2)
	Independence in toilet activity (3)	
	Concerns about post-discharge life (3)	
**Care environment**	Whether or not to renovate home (4)	Whether or not to renovate home (7)
	Location (2)	Location (6)
	Familiar place (7)	Familiar place (5)
	Relationships with familiar people (12)	Long-term care experience (6)
	Freedom in my own way of living (5)	Health condition (1)
		Presence of others who need care (1)
		Changes to life (2)
		Care and appreciation for staff (5)
**Family relationships**	Appreciation for family after being hospitalized (4)	Fulfilling the role as family (5)
	Want to help the family (5)	The patient’s intentions are unknown (5)
	Care and concerns towards the family (10)	Complying with the patient’s wishes (4)
**Disease management**	Disease recurrence/prevention (5)	Medical care needs (7)
	Response to emergency situations (5)	Early detection of the disease (1)
**Social resources and costs**	Need to continue rehabilitation (3)	Need to continue rehabilitation (7)
	Economic burden (5)	Economic burden (6)
	Services (3)	Want to avoid social withdrawal (3)
	Scope of services by service personnel (2)	Want the patient to do what he/she wants to (1)
		Worries about leaving the patient alone at home (3)
		Impression of facilities (3)
		Conditions of facilities (4)
		Difficulty of application for services (6)
		Service types (4)
		Services by service personnel (4)

() Number of codes.

In addition, we selected items for the DA that would help clarify these 5 values and that we considered to be important information from the professional viewpoint. The contents were selected to include the following: contents of long-term care insurance services, consultation services, costs, home repairs, prospects during hospitalization, family wishes, ADL, and responses to illness.

The layout of the DA was designed to enhance legibility for older adults by using large font sizes, easy-to-read fonts, and clear contrasts. Furthermore, the DA had a 2-page format printed on a single folded sheet to make it user friendly for users who may have disabilities, such as memory impairment and aphasia, and was printed on a brochure format so that it could be used with family and clinicians. *Kanji* characters and illustrations were used with preference over *hiragana* and *katakana* phonetic letters, and items that required taking memos or writing were minimized.

### 3.2. Cycle 2: Prototype

In Introduction of DA prototype Version 1, 3 discharge destinations options, that is, “home,” “residence/facility (for independent people),” and “hospital/facility (for people who need long-term care),” were listed, and how to use the DA, flow from admission to discharge, wishes and level of preparation, and the types of clinicians and distinction of their roles were described. In Facts and information, stroke, recurrence prophylaxis, long-term insurance, and features of the discharge destinations were described in detail. In Pros and cons of various options and clear expression of values and Weighing, the pros and cons of the 3 discharge destination options were printed next to the options to enable easy comparison as they related to the values that were commonly observed in older stroke patients and their families. Furthermore, a scale of “Not important 1 point” to “Very important 5 points” was used and placed vertically next to each item so that the level of importance could be compared easily. Items were added to Checking preparedness for decision-making to identify anybody other than the respondent who would help with their decision-making and who they wanted to do it with. The DA prototype Version 1 was completed on November 17, 2017, and the development group conducted a discussion to confirm that it met all the minimal criteria, that is, 12 items of the IPDASi.

### 3.3. Cycle 3

To be classified as a DA according to the 6 qualifying criteria of the IPDASi in the comprehensibility test (Table 4), all criteria must be met. The only criterion that all respondents said they met was “Available options.” In particular, 2 respondents (18.2%) answered no for “positive characteristics (pros),” “negative characteristics (cons),” and “experience of the consequence of the options.” The 6 certifying criteria of the IPDASi must be answered by “Applicable” or higher on the 4-level scale of evaluation to meet the certifying criteria. The only items for which all respondents answered “Applicable” or higher were “Production or publication date” and “Information about the funding source used for development.” The certifying criteria were met the least for “Information about the levels of uncertainty associated with event or outcome probabilities,” with 8 respondents (72.7%) answering that it was not applicable, and 2 respondents (18.2%) answering that it was not at all applicable. The outcomes of the usability test (Table 5) of evaluation by component of the DA draft 1) ~ 5) were “Normal” or above for 90% of the respondents or higher for almost all items. Further, ≥80% respondents gave good evaluations for “understandable language,” “length,” and “content bias.” However, respondents gave the lowest evaluations for the item on “clarity of how the DA should be used.”

**Table 4:** The result of comprehensibility test (N = 11).

	No.	Yes	No		
**Qualifying criteria**	1	10/11	1/11		
	2	10/11	1/11		
	3	11/11	0/11		
	4	9/11	2/11		
	5	9/11	2/11		
	6	9/11	2/11		
		Not at all	Not applicable	Applicable	Very applicable
**Certification criteria**	7	0/10	3/10	5/10	5/10
	8	0/11	1/11	3/11	7/11
	9	0/11	0/11	2/11	9/11
	10	2/11	1/11	4/11	4/11
	11	2/11	8/11	1/11	0/11
	12	0/11	0/11	2/11	9/11

Number of respondents/total number of respondents. No. 7 was unanswered by one.

**Table 5:** The result of usability test (N = 20).

	No.	Not at all	Somewhat	Fairly	Very much
**Designing**	1	0/20	1/20	15/20	4/20
	2	7/20	10/20	2/20	1/20
	3	0/20	7/20	9/20	4/20
	4	0/20	5/20	11/20	4/20
	5	0/20	9/20	7/20	4/20
	6	0/20	5/20	6/20	9/20
	7	12/20	6/20	1/20	1/20
		Bad	Normal	Good	Very good
**Acceptability**	8	1/20	10/20	8/20	1/20
	9	2/20	5/20	9/20	4/20
	10	0/20	3/20	12/20	5/20
	11	1/20	8/20	7/20	4/20
	12	1/20	5/20	9/20	5/20

Questions 2 and 4 are reversed, number of respondents/total number of people.

DA = decision aid.

In addition, in the free response section, the need for a table of contents, ingenuity to make it easier for patients and their families to imagine post-discharge daily life, weak expression of pros and cons, layout font, font size, and colors used were pointed out. There were also requests for providing additional information on the per-month costs of living in facilities and the types and roles of staff involved during hospital stay or after discharge. Furthermore, other respondents provided opinions such as “I believe that I will be able to make the best judgment for myself with staff support and explanations based on this DA,” “Wouldn’t the gap between values and reality cause problems?,” and “It would be hard to just be handed the DA and read and understand it by myself, so it would be better to look at it while somebody is explaining it to me.”

### 3.4. Steering

To clarify the methods of use, an explanation on the instructions for use was added to the DA. The table of the pros and cons of the options was reorganized and improved. As the options were difficult to differentiate, the 2 options were named “Same place as before admission” and “Different place from before admission.” To make it easier for older adults to understand, the layout, organization, and terms used for the information were further modified into succinct and easily comprehensible expressions. Moreover, home renovations were especially important for people who wished to return to their homes to recover, so it was placed as a separate item to clearly present the values. Finally, a DA based on the 6 values of “ADL,” “Services and fees,” “Emergencies,” “Family support,” “Environment,” and “Home renovation” was created.

The DA that was revised again was distributed to one of each of the following persons: an older stroke patient, family member, physician, nurse, PT, OT, and MSW. They were then asked to check the entire document. However, only minor additions and revisions of wording were needed, and the DA was ultimately developed as a 12-page, A4 paper-sized document (Table 6).

**Table 6:** Comments and revision regarding the prototype content.

Items	Comments	Revisions
**Cover**		
• Explanation about subjects		
• Explanation about the objectives and methods of use	• A Table of Contents is needed to facilitate understanding of the overall flow.	• A Table of Contents and a transitional sentence leading to the next section were added about the methods of use.
**1. Introduction**		
• Post-admission administrative procedures and schedules		
• Presenting the 3 options of “home” or “residence/facility” for independent people, and “hospital” or “facility for people who require long-term care	• It is necessary that the flow consists of deciding how they want to live first, then deciding on the place to do it.	• The options were placed after level of preparedness for decision-making
• Level of preparedness for decision-making (before reading the DA)		
• Explanation about the roles of various multidisciplinary clinicians	• Different experts are involved between pre- and post-admission	• The explanations of the various experts were separated between before and after admission.
**2. Providing information**		
• Explanation about brain stroke	• The font is small, there are too many colors used, there are insufficient margins	• Font size was enlarged, the colors used were reduced, and margins were inserted.
• Explanation about long-term care insurance	• It is better to explain more about life after stroke than about stroke itself.	• Text was changed to information about long-term care insurance services, early detection of recurrence, and measures and responses in emergency situations
	• There is little uncertain information provided	• Use the expression “it is said that ….is important” to increase the amount of uncertain information provided.
**3. Comparison of the pros and cons of each option and clarification of values**		
• Explanation about “Home”		
• Explanation about the details of long-term care insurance services		
• Explanation about “home/facility (for independent people)”	• That the word “facility” is found in both options is confusing.	• The options were revised into the following 2 options: “Same place as before admission” and “Different place from before admission”
• Explanation about “hospital/facility (for people requiring long-term care)”	• I want to know the exact monthly cost of living in a facility	• The monthly cost of living in a facility was presented in amount.
**4. Weighting**		
• For each of the 3 options, explain the details of the 5 values: “Independence in ADL,” “Environment” “Family relationships,” “Disease management” and “Social resources and fees.” • Explain the pros and cons of the 5 values	• The expressions used for the names of values are difficult. • The characteristics of the various options are stated in detail. • There is lack of explanation about the pros and cons.	• The presence or absence of home renovation was added to make 6 values. • The expressions for the values were changed into simpler words. • Explanations about the characteristics of the pros and cons were added. • The distinction between the pros and cons were made clearer and the amount of information for each was distributed more evenly. • The pros and cons were organized in a table so that it can be understood more easily. • Details about the results of the options were added.
• Comparison of the level of importance of the values for each of the 3 options	• I feel that there is a gap between the level of importance that the author believes, versus the level of importance in real life.	• I prioritized the level of importance that I currently believe in.
**5. Checking the level of preparedness for decision-making**		
• Supporters and the details of support		
• Level of preparedness for decision-making (after reading the DA)	• A box for writing out the details of the ultimate decision is necessary.	• A box for writing out the details of the ultimate decision was created.
**Back cover**		
• Citations and references related to the evidence		
• Author, date of preparation, and policies for updates		
• Sources of funds		

ADL = activities of daily living, DA = decision aid.

## 4. Discussion

This study aimed to develop a DA based on the values of older adults and their families to help older stroke patients choose where they wanted to receive post-discharge care. First, older stroke patients and their families were surveyed via an interview, multidisciplinary clinicians were surveyed via a questionnaire, and medical records were reviewed to extract the values and other data to base the DA design on. Next, a prototype was created, the IPDASi 12-item comprehensibility test and usability test were performed, and repeated revisions were made to perfect the DA. Herein, the process of development and evaluation of the DA using comprehensibility and usability tests are discussed.

First, the ultimate users of the DA, that is, older stroke patients, families, and clinicians, all repeatedly participated in the process of development. The most challenging task in Japanese healthcare settings is decision support regarding discharge destinations, which is reported to consist of adjusting the “gaps in intentions” between old older adults and their families and even medical staff.^[7]^ This can be attributed to the fact that older stroke patients are not participating much in the decision-making and that their values are instead being conveyed by their families or clinicians. Although their participation in the process of development is important, it is often difficult to involve frail older adults in practice, and it is recognized that there are many disagreements as to which stage is the most suitable for them to be involved in.^[27]^ However, Dugas et al^[11]^ stated the importance of involving the patients in the process of development to avoid stigmas and explained how it is helpful for highlighting the problems in the real world. The present DA incorporated the perspectives of older stroke patients, their families, and clinicians in the process of development, and the participation of the older stroke patients themselves is noteworthy, particularly in Japan. Japanese people value communication in indirect ways, such as the use of metaphors and hinting,^[28]^ and believe that they do not need to express themselves to be understood by others, for example, older adults themselves often forego expressing their wishes clearly. Therefore, getting older stroke patients involved and having them participate repeatedly in the development allowed creating a DA that promotes mutual understanding with families and clinicians and is easy to accept for all parties. Moreover, having multidisciplinary clinicians participate repeatedly in the process of development was also notable. Discharge care in multidisciplinary teamwork in Japan is reported to be challenging due to disparities between staff in their discharge support skills and the lack of understanding of the functions and roles of partner facilities.^[7]^ In addition, it has become clear that physicians, nurses, rehabilitation staff, and welfare staff all share this feeling of difficulty.^[29]^ In this study, there was no difference in the answers of individuals with different occupations in the questionnaire survey for clinicians. Further, no particular discrepancy in the subsequent evaluation and confirmation was observed. This suggested that having a clear goal of decision-making on discharge destination and sharing opinions and compromising in each scenario would also increase the mutual understanding of various professional roles.

Second, the DA was developed based on the common values of older stroke patients and their families. The values related to discharge destination decision-making common to older stroke patients and their families in this study were “ADL,” “Services and fees,” “Emergencies,” “Family support,” “Environment,” and “Home renovation,” showing that there were actually many commonalities between the values of older stroke patients and their families. Garvelink et al^[14]^ reported that “friends, family, or community members’ support,” “home renovations,” and “fees that the home, daily life, and services will cost” were important elements of frail older adults’ decisions on where to receive care. Murray^[24]^ reported “Privacy,” “Self-management,” and “Relationships with family and pets” as important values for terminal-phase older adults in deciding where to receive additional care. The outcomes of our study were similar in that our respondents valued connections with family and the community. The damage caused by relocation is especially serious for older adults and is reported to comprise physical, mental, and social damages.^[30]^ For older adults who have lived for a long time in environments that they have become acclimatized to, the discharge destination can have a greater impact than the change of location per se, so we considered that minimizing change and preventing isolation would ensure the smoothest path for the older stroke patients and their families to the lifestyle they envisioned. Furthermore, as a characteristic of the present case, ADL and responses to emergencies were also viewed as important. ADL^[31]^ and medical acts^[32]^ have already been known to affect older stroke patients’ discharge destination, which were consistent with the findings from the clinicians’ viewpoint. Niiyama^[33]^ reported older stroke patients’ experiences related to changes to self-image and needing to depend on the help of others, such as “unable to move,” “receiving the help of others,” and “living with disability.” When suffering from a stroke, the anxiety that the acquired ability is impaired and may recur has a great impact on patients’ post-discharge life plans, suggesting that it is necessary to consider the characteristics of the disease.

Third, although the majority of the respondents left positive responses in the evaluation of the internal validity of the DA, the evidence for the older stroke patients’ discharge destination remains vague. Evidence supporting that home or a facility is better for older adults’ convalescence, either in Japan or abroad, is lacking.^[26]^ Therefore, IPDASi evaluations for positive and negative characteristics of the options, experience of the consequences, and information about the levels of uncertainty associated with event or outcome probabilities were low, even after identifying the factors associated with discharge destination from the medical record review. Furthermore, a DA that guides a rational decision-making process needs to present information on the positive and negative evidence for the options equally. However, our findings also suggested that the evaluators of this DA were not familiar with the DA itself as there are no established DA methods for Japanese healthcare settings. It is difficult for older adults who have suddenly suffered a stroke to read, understand, and make decisions based on the DA alone. Therefore, by using the DA, patients can organize evidence-based information that suits their values and make more satisfactory decisions in a short hospital stay. In the future, it is hoped that DAs will be used together with families and specialists. The quality and quantity of information and how to utilize it also warrant further research.

A 12-page, A4-sized DA based on the 6 common values between older stroke patients and their families, that is, “ADL,” “Services and fees,” “Emergencies,” “Family support,” “Environment,” and “Home renovation,” was developed, and its internal validity was confirmed. The DA allows patients to sort out evidence-based information that matches their values and make more satisfying decisions within a short hospital stay. In the future, it is necessary to verify the effectiveness of the DA in clinical practice.

### 4.1. Study limitations and future prospects

There were several limitations to this study. First, because feedback from so many different people, such as older stroke patients, their families, and various clinicians, was needed in the process of development, only feedback from a small number of each could be evaluated. Furthermore, as there were little data on where older adults receive post-discharge care and this data were supplemented by a review of medical records from a single institution, the data were not versatile and lacked generalizability. However, to the best of our knowledge, this is the first study that involved the participation of families, clinicians, and, most remarkably, older stroke patients themselves in the process of developing the DA from the preliminary survey of needs. In future, we plan to assign participants into 2 groups, one that is provided this DA and one that is not, to evaluate it in a randomized controlled trial.

## Acknowledgments

We express our sincerest gratitude to the older adults, their families, and clinicians in the hospital for their participation, which made this study possible. This work was supported by KAKENHI Grant-in-Aid for Young Scientists (B) (15K20759) and Grant-in-Aid for Scientific Research (C) (19K11244). The authors of this work have nothing to disclose.

## Author contribution

YA designed the study and drafted the manuscript. KN provided advice on the analysis and study overall, as well as offering suggestions. All authors have consented to publication and have checked the final document.

**Conceptualization:** Yoriko Aoki, Kazuhiro Nakayama.

**Data curation:** Yoriko Aoki, Kazuhiro Nakayama.

**Formal analysis:** Yoriko Aoki, Kazuhiro Nakayama.

**Writing – original draft:** Yoriko Aoki.

**Writing – review & editing:** Yoriko Aoki.
